# 3D printing of a novel dental implant abutment

**DOI:** 10.15171/joddd.2018.047

**Published:** 2018-12-19

**Authors:** Les Kalman

**Affiliations:** Assistant Professor, Restorative Dentistry, Coordinator, Dental Outreach, Schulich School of Medicine & Dentistry, Western University, London, Ontario, Canada

**Keywords:** Dental implant, implant abutment, prosthodontics

## Abstract

Implant-supported crowns remain an ideal treatment option for the replacement of a missing tooth. The provisionalization phase remains a critical step between surgery and final crown placement, guiding soft tissue healing and providing esthetics and function. Although there are several possible pathways for provisionalization, the options are time-consuming, technically difficult and expensive, resulting in confusion and frustration for the dentist and cost and time for the patient. A novel dental implant abutment has been developed that aims to resolve the shortcomings of current abutments and the provisionalization process. 3D printing or additive manufacturing, with plastic and metal, were employed as an alternative approach for production of the prototype abutment. Scanning, computer-aided design and 3D plastic and metal printing were employed. Abutments were fabricated in MED690 VeroDentPlus and Duraform 316L stainless steel, respectively. Prototypes were printed with a claimed accuracy of 16 µm (plastic) and 8 µm (metal). The prototypes were qualitatively assessed for functionality by implant threading and simulated provisionalization process in a laboratory setting. The plastic prototypes were not suitable due to threading issues and material weakness. Metal prototypes tolerated artificial tooth fabrication successfully but concerns with thread pitch and accuracy remained. 3D metal printing appears to be a suitable alternative to traditionally machined implant components; however, post-production processing seems to be required. Further research is warranted.

## Introduction

Implant-supported crowns remain an important treatment option for the replacement of a missing tooth.^1-3^ The dental implant preserves the bone level,^4^ the abutment acts as the interface between the implant and prosthesis and the prosthetic tooth addresses the functional and esthetic demands of the patient.^5^ The provisionalization phase remains a critical step between surgery and final crown placement, guiding soft tissue healing^3,6^ and providing esthetics and function.^7^

Abutments vary tremendously in dimension, material and manufacturer.^8^ Although there are several possible pathways for provisionalization,^8^ the options are time-consuming, technically difficult and expensive.^9^ These issues can cause confusion and frustration for the dentist and cost and time for the patient.^7,8^

Due to the high potential for clinical failure^3,10,11^ and a demand for optimal esthetics,^12^ considerable scientific interest has been focused on refining the components and processes for predictable implant provisionals.

A novel dental abutment has been developed and patented,^13^ termed the Tempcap (Research Driven, Kilworth, Ontario, Canada), that addresses the shortcomings of current dental implant abutments. Research seems to suggest that the novel abutment provides an alternative which is a simple, efficient and effective solution for the fabrication of an provisional artificial tooth.^9^ Previous investigations have demonstrated the clinical application of the device on a temporary basis,^9^ as a long-term permanent abutment,^14^ and data seems to illustrate the potential advantageous.^15^ This report briefly explores the application of 3D printing, or additive manufacturing, with plastic and metal, to produce a novel dental implant abutment and the subsequent qualitative laboratory assessment.

## Methods

### Prototype Development

**Figure 1:**
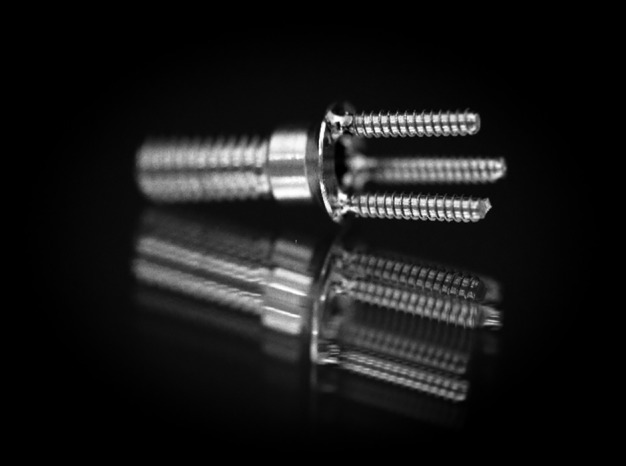
Initial prototype fabrication.

A titanium dental implant healing cap/cover screw (Implant Direct, Valencia, California) was utilized as the backbone for the prototype (Figure 1). Stabilok dental titanium retentive pins (Fairfax Dental, London, England) were utilized as retentive projections and were micro-laser welded to the healing cap by a commercial dental laboratory (LHM Dental Lab) through a detailed lab requisition. This provided the required prototype for a laboratory assessment to investigate suitability for provisional fabrication. Unfortunately, several variables (weld strength, pin failure and pin parallelism) affected the prototypes. The laboratory cost for fabrication was significant, as the components and the welding averaged about $225 CDN per prototype. Although this approach provided the initial prototype for investigation and testing, another option for fabrication was required.

Industry liaison indicated that the manufacturing of the prototype component would prove extremely difficult and costly, due to the extremely small size and exacting measurements of the component. Consistency in the quality of the pin projection was the primary concern. Additionally, manufacturing set-up costs were a risk too high for industry to even consider possible fabrication.

### 3D Printing

**Figure 2:**
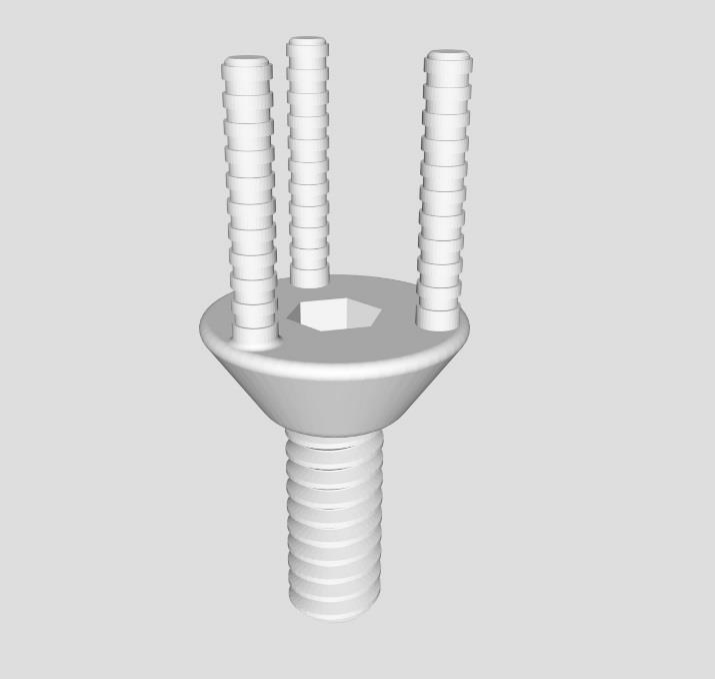
STL file format of scanned and CAD refined prototype.

**Figure 3:**
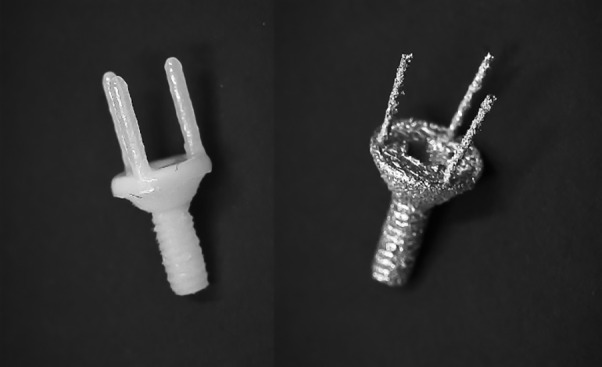
3D printed novel dental implant abutment. Left: Plastic prototype. Right: Metal prototype.

**Figure 4:**
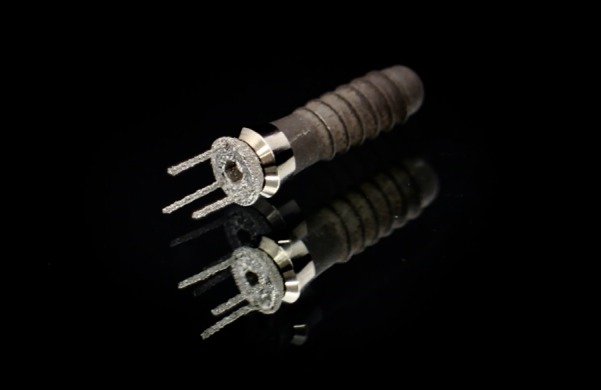
Stainless steel metal implant abutment threaded onto a Straumann dental implant.

**Figure 5:**
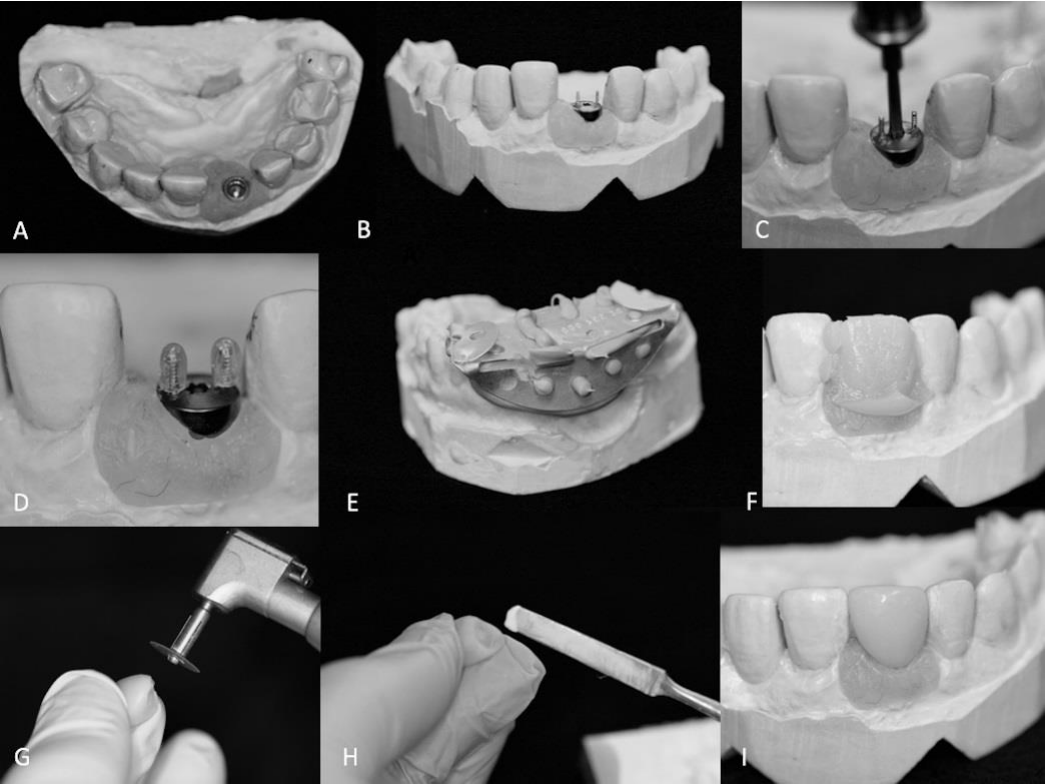
Laboratory simulated process for artificial tooth fabrication. A and B) The Tempcap abutment was placed on the master cast and C) tightened with a driver to specification. D) Impression sheaths placed on the retentive pin projections. E) A temporary matrix loaded with bis-acrylic provisional material and placed over the Tempcap, creating a F) preliminary temporary crown. G) The temporary crown is shaped and polished out of the oral cavity. H) Temporary cement is applied into the temporary crown. I) The polished temporary crown is seated onto the Tempcap abutment.

An alternative approach for abutment production was investigated. An ideal prototype, produced as mentioned above, was selected and outsourced to a commercial production facility (Cimitrex). Their team utilized scanning and computer-aided design (CAD) for refinement of the structural details of the component (Figure 2). MED690 VeroDentPlus (Stratasys, Eden Prairie, MN) material was utilized with an Objet Eden 500V polyjet printer for the fabrication of the plastic prototypes (Figure 3: left). MED690 represents a biocompatible dental material with required physical properties for the small size of the abutment. An STL file of the digitally refined component was also created. The STL file was forwarded to a separate facility (Robarts Research Institute) for additive manufacturing of the metal prototypes using Duraform 316L stainless steel and the Sinterstation Pro DM125 SLM (3D Systems Inc., Valencia, CA) (Figure 3: right). Stainless steel was selected due to its favorable physical properties and ability for sterilization. The printer applies selective laser melting (SLM) for rapid prototyping. The prototypes were qualitatively assessed for functionality by assessing if they could be threaded into an implant body (Figure 4) and if the simulated fabrication of an artificial tooth could be achieved in a laboratory setting (Figure 5).

## Results

Physical properties of each material are listed in Table 1. The prototypes were 3D-printed repeatedly with a claimed accuracy of 16 µm (plastic) and 8 µm (metal), which could not be verified due to the limitations of our equipment. Both plastic and metal prototypes could be successfully threaded into the dental implant body. The plastic prototypes could not tolerate the process of artificial tooth fabrication, as the abutment fractured repeatedly. Additionally, the plastic abutments had difficulty threading into the implant and fractured at a very low torque. The metal prototypes tolerated the process of artificial tooth fabrication successfully without any structural issues. Insertion of the metal abutments into the implant body was not ideal, with looseness and inability to achieve required torque.

**Table 1:** Select physical properties of 3D printing/additive manufacturing materials

**Property**	**Plastic (MED690 VeroDentPlus)**	**Stainless Steel (Duraform 316L)**
**Tensile Strength**	54−65	500−600
**Elongation at Break (%)**	15−25	40−60
**Modulus of Elasticity (MPa)**	2200−3200	190000

## Discussion

A novel dental abutment was fabricated, digitally scanned and refined in CAD and 3D-printed in both plastic and metal. The digital workflow has shown the capability to design and fabricate minute dental implant components. This form of plastic was too weak of a material for the application, while the stainless steel withstood the simulated provisionalization process.

Prototype fabrication remains critical in medical device research, especially in the dental implant space, and presents a significant challenge. The digital approach, utilizing scanning, CAD and 3D printing/additive manufacturing, seems to provide an alternative to traditional machining, potentially providing a cost-effective and efficient approach to device fabrication. The initial traditional prototype fabrication cost was roughly $225 CDN/unit. The approach required several weeks, to acquire the materials and for weld completion, with an approximate success rate of 50% of a utilizable prototype. In contrast, the digital design and additive manufacturing of one metal prototype required about a week, with cost at roughly $13/unit and a success rate of about 85% for a utilizable prototype. Additionally, 3D metal printing of complex structures can be produced repeatedly with accuracy, detail and with the desirable physical properties. Improvements in the process would include post-processing, especially as related to thread pitch and accuracy.

Further research is underway in collaboration with the Advanced Medical and Dental 3D Metal Printing Solutions Centre (ADEISS) at the National Research Council. Additional refinements in design, and printing in titanium 6-aluminum 4-vandium are planned utilizing a Renishaw metal printer (Gloucestershire, United Kingdom). Prototypes will be compatible with the most common Straumann (Straumann, Zurich, Switzerland) dental implants. Laboratory testing will determine failure strengths and a clinical assessment is planned.

The provisionalization phase in implant dentistry remains an important dental implant procedure. A novel abutment has been developed and investigated that seems to offer an alternative for provisionalization. Digital scanning, computer-aided design and three-dimensional metal printing/additive manufacturing have been explored as an alternative approach that seems to provide an accessible, affordable and efficient pathway for prototype fabrication.

## Acknowledgments

The author thanks Allen Xian and Drs. Holm and Holdsworth for their contribution. The research was supported by the Schulich School of Medicine and Dentistry Summer Research Studentship, Dentistry Canada Fund and the Ontario Dental Association. Thanks to our industry partners, Research Driven and Robarts Research Institute, for their assistance.

## Competing interests

The author has a financial affiliation with the subject material. The author is the co-inventor of the device and president of the company, Research Driven that maintains the intellectual property.

## Ethics Approval

Not applicable.
